# Creative Long Covid: A qualitative exploration of the experience of Long Covid through the medium of creative narratives

**DOI:** 10.1111/hex.13602

**Published:** 2022-09-23

**Authors:** Mark Pearson, Prerna Singh, Heike Bartel, Paul Crawford, Gail Allsopp

**Affiliations:** ^1^ School of Health Sciences University of Nottingham Nottingham UK; ^2^ Policy, Research and Campaigns Department Royal College of General Practitioners London UK; ^3^ School of Cultures, Languages and Area Studies University of Nottingham Nottingham UK; ^4^ Department of Clinical Policy Royal College of General Practitioners London UK

**Keywords:** health humanities, Long Covid, narrative, qualitative

## Abstract

**Background:**

Healthcare is witnessing a new disease with the emergence of Long Covid; a condition which can result in myriad symptoms, varying in frequency and severity. As new data are emerging to help inform treatment guidelines, the perspectives of those living with Long Covid are essential in informing healthcare practice. The research aimed to collect the narratives of people living with Long Covid to better understand the lived experience of this condition. In attempting to narrate complex or traumatic experiences the arts and humanities can offer alternative ways of expressing embodied narratives, representing rich sources of meaning. Therefore, the research specifically sought to elicit creative expressions from participants with lived experience of Long Covid.

**Methods:**

Data were collected via an online repository where participants could submit their pieces of creative writing. Data were collected between August 2021 and January 2022 and a total of 28 submissions were received from participants. These were mostly written creative narratives. However, a small number were submitted as audio or video files of spoken word poetry or songs. Data collection was stopped once data saturation was achieved.

**Results:**

The submissions were subjected to thematic analysis and five themes were generated. These five themes are Identity, social relationships, symptoms, interaction with healthcare systems and time. The results provide an insight into the experience of Long Covid as detailed by the participants' creative narratives.

**Conclusion:**

The results from this study provide a unique insight into the lived experience of Long Covid. In relation to clinical practice, the results suggest that adjustment reaction and loss of sense of self could be added as common symptoms.

**Patient and Public Contribution:**

Before undertaking the research, Long Covid community groups were contacted to discuss the potential value of this study and it was widely supported. One of the leading Long Covid support groups was also involved in disseminating information regarding the project. As part of ongoing work within this project, members of the team are actively disseminating the results within Long Covid communities and seeking to develop arts‐based workshops specifically for people with Long Covid.

## 1 INTRODUCTION

As described by Atherton et al.,^1^ healthcare is ‘witnessing a new disease’ with the emergence of Long Covid. Although debate remains in relation to the terminology associated with this condition,^2^, ^3^ the term Long Covid can be considered an umbrella term for clinical presentations that can be myriad in nature, severity and duration of symptoms. As clinical guidelines continue to be developed,^4^, ^5^ and specialist healthcare pathways established,^6^, ^7^, ^8^ the narrative insights gained from those who have experienced Long Covid reflect a complex collection of symptoms, often unlike anything which had been experienced previously.^9^

The importance of understanding the phenomenological interpretation of individual experiences is acknowledged both within research and healthcare practice.^10^ As part of a broader narrative turn in recent decades, narratives are considered essential to the construction and reconstruction of the self; helping individuals to make meaning of their experiences and shape their identity.^11^, ^12^ These narratives are diverse, highly complex, linear, nonlinear, often creative, and importantly, can deliver vital information to inform clinical practice, give healthcare providers a greater understanding of the illness experience. The stories people tell are attempts at ‘world making’,^13^ and valuable tools for articulating and understanding life‐affecting illness experiences such as Long Covid.

In general, narratives about illness experiences have been recognized as essential components in a process, providing healthcare practitioners with insights into the context, meaning and lived experience of the individual.^14^ However, this process of narration is often complicated,^15^ especially following what might be considered the biographical disruption resulting from chronic or acute illness.^16^ At such times, the act of narration is often orientated towards attempting to make meaning of the situation and understand why the illness has occurred, what caused it and how can it be addressed.^17^

In attempting to narrate such experiences the arts and humanities can offer alternative ways of expressing embodied narratives, representing rich sources of meaning.^18^, ^19^ Moreover, the creation of art such as poetry, creative writing and music may offer a way to express trauma‐laden narratives which thus far have remained unsayable.^20^, ^21^ In attempting to support the narration of the unsayable, art can support in the process of discovering and meaning‐making which can be essential at times of ill health.^22^, ^23^, ^34^ Therefore, this paper will apply an interdisciplinary approach that bridges medicine, arts and the humanities to analyse arts‐based patient narratives about Long Covid.

## 2 METHODS

### 2.1 Design and approach

The research adopted a narrative methodology, prioritising the narratives of individuals as a way to understanding their lived experience Frank^25^ described illness as a ‘call for stories’ both in the sense of a need to describe the experience to others but also as a process of re‐orientating and repairing the damage done by illness to the sense of who the person is and where they are going. Moreover, for healthcare professionals, individual or shared narratives can offer unique insights and enhance clinical practice by narrowing the distance between practitioner and service user.^26^ Narrative research is often a multidisciplinary undertaking^27^ and this is reflected in the diverse nature of the research team within this project. The research team included academics and clinical practitioners from background in health sciences, medicine and the humanities. The research was granted approval by the ethics committee within the School of Cultures, Languages and Area Studies at the University of Nottingham.

Narrative research should seek to engage participants in the process of storytelling,^28^ and this study sought to facilitate that storytelling process by establishing an online repository where stories could be collected and shared with the researchers. The opportunity to participate in the research was disseminated widely through social media and also posted within specific online forums dedicated to lived experiences of Long Covid. In an advertisement for the research, participants over the age of 16 were asked to submit their creative writings about their personal experiences of Long Covid, be it as a directly affected patient, as a carer or other.

The format of the online repository was developed in keeping with the values and practice underpinning narrative methodology,^29^ and only contained one prompt which was ‘Please can you use the space below to paste/write your creative written work’. These included short stories, poems, letters, diary entries or pieces of prose, all of which could be submitted directly into the online repository. Participants were also provided with the option of emailing the research team directly if they wanted to share something which was not textual, such as an audio recording or song. Data were collected between August 2021 and January 2022 and a total of 28 submissions were received from participants. These were mostly written creative narratives. However, a small number were submitted as audio or video files of spoken word poetry or songs.

Data saturation is complex and difficult to define,^30^ especially in relation to arts‐based research, due to the potentially ambiguous nature of creative submissions.^31^ However, this was felt to have been achieved once the codes produced from the data stabilized and no new themes were emerging from the data.^32^ Moreover, the researchers felt that sufficient data had been collected to develop a robust understanding of the issues raised by participants.^33^

Written consent was collected from all participants in keeping with best practices in online research.^34^ All participants were provided with the participant information sheet as part of accessing the online repository, they then were required to click several boxes to indicate that they had read and understood the information and were happy to consent to take part in the research.

### 2.2 Analysis

All the data were first organized into text format. For those pieces which were submitted as text, no changes were made to the documents. However, for those pieces which were submitted as audio files, such as a song, these were transcribed to ensure that they could be analysed consistently, alongside the other submissions.

The data were then subjected to thematic analysis following the framework of Braun and Clarke.^35^ The first stage of this analysis is to familiarize oneself with the data and this was done as a whole team who met to review the data. During this first meeting, the research team each read aloud a submission. The team then discussed the submissions, and their experiences of reading or listening to these creative narratives. This reflexive process was particularly significant during this study, due to the interdisciplinary nature of the research, and thus reflexive practice can provide opportunities for the exploration of different perspectives and underlying assumptions.^36^

Following this preliminary meeting, the initial coding of the data was undertaken by two members of the research team who analysed the data independently before meeting to review the codes that each had identified. These codes were then shared and refined into themes through a process of ongoing discussion with the wider research team.

## 3 RESULTS

The results of the analysis identified five themes, which are as follows: Identity, social relationships, symptoms, interaction with healthcare systems and time. Table 1 details each theme and the codes that are comprised within it.

**Table 1:** Themes and codes

Themes	Codes
Identity	Loss of old identity
	New identity and future self
	Externalization of Long Covid
	Career disruption
Social relationships	Change in role
	Social isolation
	Invalidation by others
	Support groups
Symptoms	Physical symptoms
	Cognitive symptoms
	Activities of daily living
	Variability of symptoms
Interactions with health system	Lack of answers
	Multiple appointments
	Recommended treatment inefficacy
	Loss of trust in professional advice
Time	Expected versus real recovery time
	Uncertainty about recovery
	Passage of time
	Loss of future

### 3.1 Self and identity

The notion of self and identity can be conceptualized as the evolving life story which integrates our past and imagined future.^37^ Participants spoke of the way in which the experience of Long Covid had required them to address the way in which they conceptualized themselves in relation to their illness. Several of the participants described feeling that pre‐covid they were healthy people:

Prior to Covid, I was healthy and loved my life (Participant 12)

Since that first attendance award at age 5 I've taken pride, in weathering common colds and tummy bugs, top of the class at being completely fine (Participant 25)

The resulting impact of Long Covid had therefore resulted in participants trying to reorientate themselves to their current life situation, accommodating the physical toll of Long Covid did not fit with the narrative of themselves. The creative forms of expression used allowed participants here to reflect upon elements of orientation and disorientation, order and disorder not merely on the level of content but also on the level of form. The following excerpt from the participant's 16 poems brings in the last, almost comical punchline the rhyming figure of ‘eighty’ reflecting the ‘multiple frequency’ of afternoon naps needed by a much younger person who due to Long Covid feels old beyond their years:

Take afternoon naps

Of multiple frequency,

But you're not eighty! (Participant 16)

… a loss of me, like a very close death to deal with, but this was me (Participant 7)

In considering the temporal relationship between narrative and identity, perceiving oneself within a narrative with a past, present and future,^38^ participants spoke of struggling to reconcile their current sense of self with that which they had experienced before Long Covid:

I think I have forgotten what normal life used to be (Participants 18)

Long Covid robbed me of my life and dreams, I am merely a ghost that can haunt the fringes of my former life (Participants 12)

The metaphor used by participant 19 of a frayed and tattered flag that is merely a remnant of the former intact fabric adds a very evocative and sensuous angle to their narrative and speaks to the power of metaphor in patient narratives^39^, ^40^, ^41^:

There are still remnants of the old me, they're frayed and tattered like a flag by the sea (Participant 19)

Participants also speculated about the future and whether this shift in identity might represent something more permanent:

Will the old me come back?

Or will I always be this way? (Participant 3)

Participants also spoke of their future and the way in which Long Covid had influenced their perception of this future. For many people

My life it slipped away. My work, my future, core beliefs, Lost to this disease (Participant 8)

It also seemed to be triggering existential questions, in relation to why they were experiencing these symptoms when others were not having these experiences.

Why me, what have I done to deserve this life‐changing virus? (Participant 7)

What am I doing here?

Is this me?

A new me? (Participant 3)

Who am I now? I'm not the same (Participant 8)

Participants also spoke of Long Covid in a variety of externalized terms. The externalization of illness is a therapeutic process of locating the illness or condition as external to the sense of self. This reinforces the notion that ‘the problem is the problem, the person is not the problem’.^42^ Participants often differentiated the virus from their sense of self and identity:

Long Covid sits and laughs at me, reminds me of the past (Participant 8)

I've not improved, Long Covid doesn't want me to (Participant 19)

Additionally, some participants directed their creative piece towards Long Covid as an agent, as if taking the opportunity to communicate directly with the virus:

I can't talk or walk without you making me short of breath (Participant 5)

### 3.2 Social relationships

As humans are social beings, our social relationships and networks provide not only meaningful interpersonal contact but also help to foster and support our stable sense of self.^43^ Following developing Long Covid, participants spoke of the changes in social roles as a result, with some commenting that traditional caring relationships had now been reversed:

I have had to move home to live with my mum, who has now become my support (where as previously I would support her emotionally) (Participant 12)

Parents sleeping, kids cooking, roles reversed for better or worse (Participant 6)

More broadly, participants identified changes in social routine as a result of Long Covid, especially in relation to the frequency or quality of social contact. As social identification to particular groups or categories has significant impacts for well‐being across a range of illnesses or disabilities,^44^, ^45^, ^46^ there is the potential for people to experience a negative impact on their well being as they are no longer able to engage with their social groups in the same way, and thus may feel a reduced sense of belonging:

Able to exercise before work if I fancied it and was non‐stop socialising, to say it is a slight change to what I know is a huge understatement (Participant 10)

I feel as though I am losing my friends, and whilst they understand that I am ill, the lack of attendance means you inevitably drift away (Participant 12)

Some participants also spoke of concerns regarding the way in which Long Covid, and the impacts of Long Covid, may or may not be validated by those within their social networks. This seemed to be especially salient during the early stages of the pandemic when little was known about the condition:

Aren't you better? I often get asked

Friends and family are aghast,

When I say no, I've not improved (Participant 19)

Can't they see that they don't feel the same?

Why is it so hard to understand?

Why is it so difficult to explain? (Participant 3)

There was a sense also of the way in which the symptoms of Long Covid were not acknowledged or validated by wider society or specific organizations, such as those within health and welfare. This was perhaps particularly relevant in relation to individuals not being able to work and therefore perceiving judgement due to inactivity or lack of work^47^:

please

please believe me

when I say this isn't something I normally do

but I just can't seem to focus on this literature review (Participant 25)

…lived in hell a body tortured. Refused PIP as there was nothing wrong with me, I was been silly, Long Covid didn't exist… (Participant 11)

Touch of anxiety they say, THEY DONT EVEN KNOW ME (Participant 15)

Participants also highlighted the nature of Long Covid as potentially an invisible illness and the social difficulties which might arise as a result:

What would you say, to the people who say

Oh yes I've been tired too

Don't worry I forget things too

I can't find words when I'm tired either

You look so well

You look better (Participant 3)

However, conversely, participants also spoke of the way in which they have to manage the impact of Long Covid, in particular, to shield the impacts from those they loved:

Unable to move, I lie, my child watching TV oblivious to the Covid doom lurking over his father (Participant 15)

Trying to find the strength to carry my Dad's coffin after he passed away

Helping support my Mum, holding my business together, all whilst feeling so ill (Participant 18)

As a result of the reduced social contact and perhaps a reduced sense of opportunities for authentic communication, participants highlighted the importance of peer support groups which they had joined during their experiences of Long Covid:

I'm extremely grateful to the support groups online and those people who've shared their stories already (Participant 10)

And then along came…and her Facebook group. A safe place. People who listened and helped (Participant 2)

I discovered I was not alone, new friends I have now made

I know there is always someone there, no need to be afraid (Participant 18)

One participant added the perspective of ethnicity to the topic on a personal but also a political level by weaving into their text a widely used and recognizable quotation. The text ends with

I can't breathe (Participant 28)

This is a clear reference to the last words of African‐American George Floyd after a policeman knelt on his back and neck for a considerable time in 2020 in Minnesota. The text puts the individual experience of Long Covid into the context of experiences of racial injustice in the healthcare system.

### 3.3 Symptoms

Participants described experiencing several physical and cognitive symptoms. All of these align with the symptoms listed in SIGN/NICE (2021) guidance and also reflect the most commonly reported symptoms across wider literature echoed within NICE guidance.^48^, ^49^, ^50^ Respiratory symptoms (shortness of breath), generalized symptoms (fatigue, pain) and neurological symptoms (‘brain fog’) were described almost unanimously. Other commonly expressed symptoms included cardiovascular symptoms (palpitations), ear nose and throat symptoms (loss of taste and/or smell), gastrointestinal symptoms and further neurological or psychological symptoms (loss of balance, loss of coordination, loss of concentration and difficulty speaking):

Did I mention the bruising, the hair falling out, the vibrations felt inside?

Did I mention chest pain, visits to A&E and hearing compromised?

Did I mention the constant sore throats, hoarse voice and sensitive eyes?

Did I mention my friendship with my toilet? A Long Covid/G.I. surprise! (Participant 18)

Frequently, the participants within this study described experiencing consistent and debilitating levels of fatigue:

I have been exhausted since testing positive and I'm talking utterly fatigued (Participant 10)

Take tired and exceed it by such an extent that your body fills with fatigue (Participant 21)

Shortness of breath during minimal exertion and resting states was also commonly described:

Being so out of breath even when resting has been scary (Participant 10)

All I want

to do is sleep and be able

to breathe, just breathe (Participant 25)

Head down, breathe hard, breathe out (Participant 9)

Among notable cognitive symptoms, participants reported experiencing a clouding of consciousness, often described as ‘brain fog’, as well as reduced ability to concentrate, and increased episodes of confusion:

My brain is still in such a fog that I can't concentrate or read like I could before (Participant 5)

I think I have forgotten what normal life used to be…

Not putting the kettle in the fridge or forgetting which words to say (Participant 18)

Notably, participants described the significant hindrance that their symptoms posed in their ability to carry out activities of daily living:

To shower in the morning I have to check I've got enough energy to wash my hair and deal with it afterwards. I often need to rest between showering/dressing/doing hair (Participant 10)

It's so hard, when you spend an hour opening a herbal teabag, too tired to cook dinner as your body has suddenly gone tired, or cooking dinner and you have no way to get the food to your mouth, or you use fingers (Participant 11)

The severity and the types of symptoms were described as variable from day to day:

I miss getting up and not wondering which symptoms that day will reign (Participant 18)

Every few weeks brings relief and fear

As old symptoms leave and new ones appear

One day is better, the next one is worse (Participant 19)

This made it difficult for participants to plan their weekly schedules in advance. Instead, many participants described the unpredictability of having to plan their day according to the severity of symptoms:

Everything planned around my fatigue levels and need to rest (Participant 10)

Your alliteration is

Plan, Pace, Prioritise! (Participant 16)

Externalization is a technique often employed by therapists during narrative therapy.^42^ Encouraging a person to describe their issues using an external form facilitates healing by validating the individual's experience and enables the individual to perceive their identity as being more than just an embodiment of their problem.^51^ Many participants conceptualized their symptoms as having an external form with its own sense of agency:

What is the disease? Dementor, figure of self‐hate, punishment, curse? (Participant 21)

Unable to move I lie, my child watching TV oblivious to the Covid doom lurking over his father (Participant 15)

These external concepts sometimes represented the evolving nature of symptoms over time:

Covid's a shape shifter that wants to be king (Participant 19)

like a hydra It developed new heads, kicking back escape (Participant 1)

#### 3.3.1 Interactions with the health system

As Long Covid has only recently emerged as a condition, there remains a relative lack of understanding amongst healthcare professionals. Trust and knowledge are two of the four key elements of an effective clinician‐patient relationship.^52^ Therefore, a perceived lack of knowledge in a clinician has the potential to significantly impact the therapeutic relationship. Participants expressed being aware of their doctor's limited ability to help them at the present time and the corresponding feelings of uncertainty and helplessness:

Yes I'm ok it's just I can't breathe or stand or move my feet to the music and my Doctor can't help me (Participant 2)

Docs have no answers and say, ‘Just wait and see!’ (Participant 19)

This resulted in some participants expressing a diminished sense of trust in the benefit of interactions with the healthcare system:

Will there be a treatment?

I don't trust the system anymore.

When did I become so cynical? (Participant 3)

The symptoms were different, not what they had said (Participant 4)

Appointments, disappointments (Participant 6)

A drastic increase in the number of healthcare appointments was described. Participants held mixed feelings about these. Some participants expressed frustration at having to attend multiple appointments whilst others expressed feeling gratitude for the opportunity to receive help from multiple departments:

I'm awaiting appointments with the chronic fatigue service and local physiotherapists and also need pulmonary function tests. All of this and months later I'm no further forward in how I feel. No one's fault, research takes time (Participant 10)

I have been fortunate enough to see many specialists (cardiology, neurology, rheumatology, respiratory, complex medical specialist) and have tried all available therapies, including those which are less proven and I need to purchase medications online. None of these have helped (Participant 12)

Everyday a new specialist I meet…

How long will this last—will I ever be me? (Participant 19)

#### 3.3.2 Time

Temporal constructs play an important role in the formation of chronic illness narratives. The presence of long‐term symptoms can alter the experience of temporal rhythms, resulting in a unique relationship with time that differs from those experienced by healthy or acutely unwell individuals.^53^ Participants gave notable significance to the amount of time that had passed since the onset of their symptoms, often expressing this as a number of calendar months. The imagery of changing seasons was also juxtaposed with the constancy of symptoms:

The time it ticks away

The weeks, the months and now a year

Long Covid's here to stay (Participant 8)

How long is Covid?

Happy anniversary—

12 months, ongoing (Participant 16)

Spring sap rising

Summer green gold

Autumn blaze of dying

Winter stripped cold.

A year passed by.

I'm still waiting (Participant 17)

Biographical disruption refers to the effects of a sudden event that significantly alters the existing course of an individual's life, including their external plans and internal perception of their life.^54^ Participants expressed feeling that the course of their life has been disrupted, including a sense of loss of opportunities, dreams and future:

My world is smaller

I have missed so much

I feel utterly lost (Participant 3)

My life it slipped away

My work, my future, core beliefs (Participant 8)

Little did I know I had had the last day of my active life (Participant 11)

Long Covid robbed me of my life and dreams (Participant 12)

## 4 CONCLUSION

The participant narratives align with many of the symptoms described and present in NICE national guidance.^5^ Fatigue and shortness of breath being the most common, but many others, including palpitations and neurocognitive symptoms also being reported. However, the data collected within this study suggest that some people may lack trust in the healthcare system, with participants often perceiving themselves as knowing more about their condition than the clinician working with them. This required further investigation as new healthcare pathways continue to emerge with general practitioners (GP) often playing a key role in the management of Long Covid.^55^ Kingstone et al.^56^ describe how finding the right GP is essential for people with Long Covid, emphasizing that listening, the use of empathy and understanding are a key part of the initial consultation. The relationship between a healthcare professional and an individual is well established as an essential component in any healthcare intervention and has been emphasized as crucial in supporting people experiencing Long Covid.^1^, ^57^, ^58^

This study also highlights other symptoms and experiences, common in many of the participant narratives. Loss of self, loss of identity and significant adjustment reactions to their pre Covid lives are powerful themes evident within the creative submissions. Psychological adjustments to illness can be considered as adaptive or nonadaptive as individuals try to match their capabilities to the demands of their environment.^59^ This process of adjustment is not detailed in national guidance but is key to understanding the frustration of people with Long Covid and the potential emergence of nonadaptive responses such as depression, social withdrawal and in severe examples attempting to end one's own life.^60^, ^61^ This process of adjustment takes time and is not currently listed in the most common symptoms of Long Covid and therefore may be overlooked by those looking within healthcare. Therefore, the authors suggest that adjustment reaction and loss of sense of self could be added as a common symptom to reflect the lived experience of Long Covid.

The majority of the estimated 1.3 million people living with Long Covid in the United Kingdom are between the ages of 39–65 (Office of National Statistics, 2022). This is an age group where people are usually fit and well, at the prime of their life, with the majority expected to be at work. Moreover, whilst adjustment and coping theory have often focused on the response of individuals, there is increasing recognition of the impact the individual is situated within their social context and that the changes to their life will influence those around them.^62^ Therefore, consideration needs to be given to not only the effect on the individual of Long Covid but also the impact on their social network.

## AUTHOR CONTRIBUTIONS

Mark Pearson contributed to the design and implementation of the research. He also co‐led on data analysis and led the writing of the manuscript. Prerna Singh contributed to the design and implementation of the research, co‐led the process of data analysis, and significantly contributed to the writing of the manuscript. Heike Bartel designed the study, led the development of the tool for data collection and helped to supervise the project and also contributed to data analysis and to the writing of the manuscript. Paul Crawford was consulted at various phases throughout the project, especially during the phase of data analysis and contributed to reviewing the manuscript. Gail Allsopp led the conceptualization, design and supervision of the project; and also led patient and public involvement and contributed to writing the manuscript.

## CONFLICT OF INTEREST

G. A. is the clinical policy lead for the Royal College of General Practitioners and led the RCGPs input into the NICE guidance https://www.nice.org.uk/guidance/ng188. The remaining authors declare no conflict of interest.

## Data Availability

The data that support the findings of this study are available from the corresponding author upon reasonable request.
